# Risk of SARS-CoV-2 infection in high and low-risk cohabitants, in Loures and Odivelas, Portugal: Inês Mateus Cunha

**DOI:** 10.1093/eurpub/ckac131.093

**Published:** 2022-10-25

**Authors:** I Mateus Cunha, JL Marques, IM Subtil, M Bragança Pereira, C Gabriel, C Vieira, C Cordeiro, F Abreu Gomes, L Bastos

**Affiliations:** 1 Public Health Unit, Group of Primary Healthcare Centers of Loures-Odivelas, Santo António dos Cavaleiros, Portugal; 2 Superior Nursing School of Lisbon, Lisbon, Portugal

## Abstract

**Background:**

Effective contact tracing, vaccination, and isolation of cases of SARS-CoV-2 infection and their high-risk contacts constituted measures to contain the spread of COVID-19. In Portugal, in October 2021, low-risk cohabitants were lifted the obligation to isolate. The aim of this study was to estimate the relative risk of infection for close contacts, regarding the type of close contact and being cohabitants.

**Methods:**

A descriptive longitudinal study, with an analytical component was performed. Sociodemographic and epidemiologic data from close contacts and confirmed cases in Loures and Odivelas, between October and November 2021, was collected from a regional database and from Trace COVID-19 platform. We performed a descriptive analysis and estimated the relative risk of SARS-CoV-2 positive test, stratified by type of contact and cohabitation, with 95% confidence level.

**Results:**

We identified 200 confirmed cases and 428 people who were close contacts, corresponding to 502 different close contacts (59 people had contact with more than a case). From 502 close contacts, 268 were classified as low-risk and 230 as high-risk. Full time cohabitation was present in 310 of close contacts. Between contact tracing day and the next 4 weeks, 58 (10.9%) of close contacts tested positive. Risk of high-risk contacts testing positive was 2.7 [1.5-4.6], compared with low-risk contacts. Risk of cohabitants testing positive was 3.5 [1.6-7.7], compared with non-cohabitants. Risk of a high-risk cohabitant testing positive was 2.2 [1.1-4.4], compared with low-risk cohabitants. There was no higher risk of high-risk cohabitants testing positive compared with high-risk non-cohabitants. Same was true for low-risk cohabitants and non-cohabitants.

**Conclusions:**

These results allow us to understand how to better stratify close contacts and apply isolation measures, according to the risk of testing positive. Further studies should be developed to assess the impact of other variables.

**Key messages:**

• We identified an increased risk of testing positive in high-risk contacts, and in cohabitants.

• Cohabitants could be stratified regarding being high or low-risk, with different measures being applied.

